# Diabetes mellitus does not increase the risk of knee stiffness after total knee arthroplasty: a meta-analysis of 7 studies including 246 053 cases

**DOI:** 10.1186/s43019-019-0004-4

**Published:** 2019-07-17

**Authors:** Christopher Jump, Rayaz A. Malik, Anoop Anand, Charalambos P. Charalambous

**Affiliations:** 1 Department of Orthopaedics, Blackpool Victoria Hospital, Blackpool, UK; 2 Weill Cornell Medical College, Doha, Qatar; 3 Centre for Endocrinology & Diabetes, Institute of Human Development, University of Manchester, Manchester, UK; 4 School of Medicine, University of Central Lancashire, Preston, UK

**Keywords:** Diabetes mellitus, Knee stiffness, Arthrofibrosis, Total knee arthroplasty

## Abstract

**Purpose:**

The association of diabetes mellitus with knee stiffness after total knee arthroplasty is still being debated. The aim of this study was to assess through meta-analysis the impact of diabetes mellitus on the prevalence of postoperative knee stiffness after total knee arthroplasty.

**Methods:**

We conducted a literature search for terms regarding postoperative knee stiffness and diabetes mellitus on Embase, CINAHL, and PubMed NCBI.

**Results:**

Of 1142 articles, seven were suitable for analysis. Meta-analysis showed that diabetes mellitus does not confer an increased risk of primary or revision total knee arthroplasty-induced postoperative knee stiffness when compared to nondiabetic patients (primary total knee arthroplasty, estimated odds ratio [OR] 1.474 and 95% confidence interval [CI] 0.97–2.23; primary and revision total knee arthroplasty, OR 1.340 and 95% CI 0.97–1.83).

**Conclusion:**

There is no strong evidence that diabetes mellitus increases the risk of knee stiffness after total knee arthroplasty. The decision to proceed with total knee arthroplasty, discussion as part of the consent process, and subsequent rehabilitation should not differ between patients with and without diabetes mellitus with regards to risk of stiffness.

**Level of evidence:**

Level III (meta-analysis)

## Introduction

The aim of total knee arthroplasty (TKA) is to provide the patient with a stable, pain-free knee with a functional range of movement (ROM) that allows activities such as walking, standing from a chair, and ascending and descending stairs. To achieve this, it is estimated that up to 105° of knee flexion is required [1].

Postoperative knee stiffness is a significant, disabling complication of TKA resulting in a reduction of ROM, which significantly reduces the patient’s ability to perform activities of daily living (ADLs) and their quality of life (QoL) [2]. ROM is also an important postoperative factor affecting patient satisfaction [3].

The incidence of knee stiffness after TKA varies from 1.3 to 12% [4–6]. This large variation can be explained by the lack of uniform definition of stiffness in the literature: flexion less than 90° at 2 weeks [6] and 1 year after TKA [4]; flexion less than 85° [7] and less than 110° ROM at 6 weeks after TKA [8]. Stiffness after TKA can be limited by active physiotherapy but may also require manipulation under anesthesia (MUA), which increases the risk of revision surgery.

Many risk factors may contribute to knee stiffness after TKA: reduced preoperative ROM, prosthesis malpositioning, imbalance of flexion/extension gaps, noncompliance with postoperative rehabilitation, socioeconomic status, race, gender, and diabetes mellitus (DM) [5, 6, 8, 9]. In particular, Scranton reported that 85% of TKA patients with stiff knee had DM [7].

DM currently has a prevalence of over 422 million worldwide [10]. The prevalence of DM in patients undergoing TKA is 12.2%, and with an increasingly elderly population, the incidence of DM as well as osteoarthritis (OA) is increasing [11]. Patients with DM are considered to have greater overall complications and higher revision rates after TKA [12]. One in three patients with frozen shoulder has DM and 13% of diabetic patients develop frozen shoulder [13]. It is unclear if DM affects all joints equally or whether it affects certain joints more commonly.

The purpose of this meta-analysis is to establish the risk of postoperative knee stiffness in diabetic patients after TKA from the current published literature in order to provide surgeons with an evidence base for guiding them as to the likelihood of suffering postoperative stiffness. Increasing current knowledge on the prevalence of postoperative knee stiffness may also help to set realistic goals of postoperative ROM and allow more rigorous physiotherapy to reduce the risk of joint stiffness. We hypothesize that DM increases the risk of postoperative knee stiffness after TKA.

## Methods

A literature search was conducted on October 31st, 2018 using Embase, CINAHL, and Pubmed NCBI (National Centre of Biotechnology Information). The search terms used were ‘knee AND arthrofibrosis AND diabetes’, ‘knee AND stiff AND diabetes’, and ‘knee AND stiffness AND diabetes’. No restrictions were applied to the date of publication or language. Ethical approval was not required. The study was conducted and meets the ethical standards as per the recommendations by Padulo et al. [14].

The search returned 1142 articles. The titles and abstracts of these articles were reviewed. Studies were included if they identified the prevalence of knee stiffness after TKA in a diabetic population. Case reports, series, duplicated articles, and reviews were also excluded. The studies had to define what they considered as knee stiffness. Essentially studies included compared stiffness in diabetic and nondiabetic patients after TKA. Studies including primary and revision TKA were included. When performing our analysis, we looked at the number of TKAs performed rather than the number of patients. The Methodological Index for Nonrandomized Studies (MINORS) assessment was used to assess included studies for bias [15].

### Statistical analysis

A random-effects model was used to perform meta-analysis. Confidence intervals (95%) and summary odds ratios (OR) were calculated. Heterogeneity was assessed using tau^2^, I^2^, Q, and *P* values. The data were analyzed using Comprehensive Meta-analysis version 2 (Biostat, Englewood, New Jersey, USA).

## Results

**Fig. 1:**
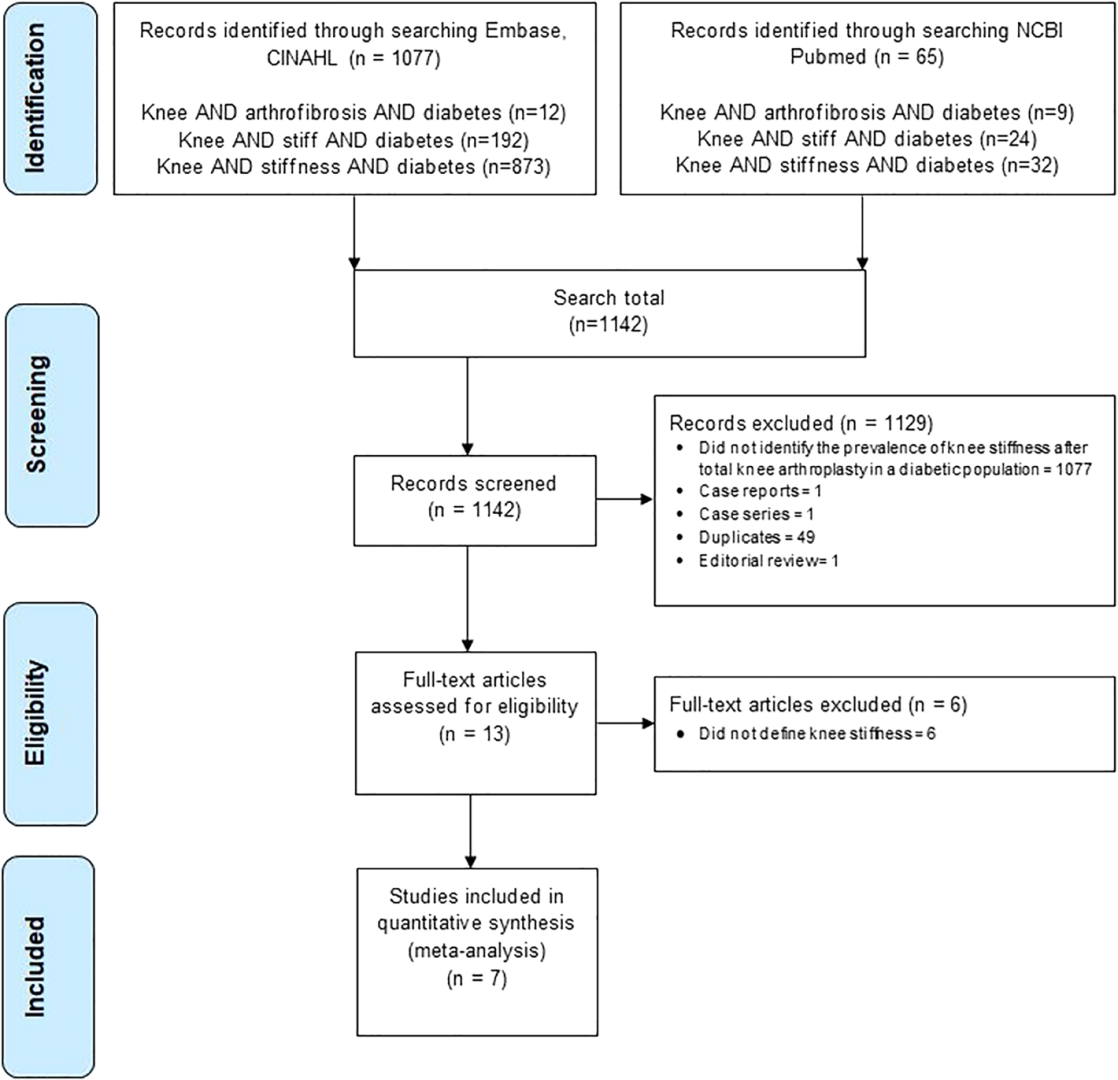
Literature search results (Preferred Reporting Items for Systematic Reviews and Meta-Analyses [PRISMA] flow chart)

Our initial search identified 1142 articles (Fig. 1). Seven studies were included for analysis [4, 8, 12, 16–19](Table 1). Patients in these studies with and without DM were treated similarly with regard to postoperative physiotherapy. Six studies presented data on primary TKA. One study included revision TKA patients [19].

**Table 1:** Summary of studies identifying knee stiffness after total knee arthroplasty in populations with diabetes mellitus

Study	Population	Aim	Definition of stiffness	Study design	Conclusion	Number	Prevalence
Cartwright-Terry et al. [18] 2018	UK	To assess the functional outcome of manipulation in patients after knee replacement and to investigate potential predictive factors for stiffness	MUA required postoperatively. Decision based on symptoms, functional requirements, and clinical examination. No formal degree of flexion was used	Nested case–control	Patients with DM more likely to require manipulation	138 TKAs Stiff group: Total number = 69 Number of DM = 10 Control group: Total number = 69 Number of DM = 3	Percentage of stiff group with DM = 14% Percentage of control group with DM = 4%
Clement et al. [17] 2018	UK	The primary aim was to compare the outcome (WOMAC, Short form (SF)-12, and satisfaction) of patients with increased symptoms of stiffness 1 year after TKA with those who had no change or improvement in symptoms. The secondary aim was to identify independent predictors of increased symptoms of stiffness 1 year after TKA	Patients who had a worse or negative (1 year after TKA compared to preoperatively) change in the WOMAC stiffness score were defined as the increased symptoms of stiffness group. This group was compared to those who had no change or improved symptoms at 1 year	Retrospective cohort	DM significantly predictive of increased stiffness 1 year after surgery	2589 TKAs Stiff group: Total number = 129 Number of DM = 27 Control group: Total number = 2460 Number of DM = 338	Percentage of stiff group with DM = 20.9% Percentage of control group with DM = 13.7%
Dowdle et al. [19] 2018	USA	To determine the incidence of MUA after revision TKA, to better define the modifiable and non-modifiable postoperative risk factors associated with postoperative stiffness after revision TKA, and to determine the timing of MUA for postoperative stiffness	MUA performed postoperatively	Case-control	DM did not increase the risk of MUA after revision TKA	5414 TKAs Stiff group: Total number = 96 Number of DM = 41 Control group: Total number = 5318 Number of DM = 2181	Percentage of stiff group with DM = 42.7% Percentage of control group with DM = 41%
Issa et al. [8] 2015	USA	To evaluate the effect of various (1) demographic factors, (2) comorbidities, and (3) knee-specific factors on the frequency of MUA, which was used as an indicator of knee stiffness after a primary TKA	Need for MUA and < 110° of ROM at 6 weeks after TKA with no recent gains after physical therapy	Case-control	DM was associated with an increase in frequency of MUA after TKA	3182 TKAs	Odds ratio of DM to non-DM having to have MUA: 1.72 (1.02–2.31), *p* = 0.0311.
Pfefferle et al. [16] 2014	USA	To test the null hypothesis that there is no increased rate of postoperative stiffness requiring an MUA within 90 days of a TKA between groups stratified by race, gender, nicotine dependence, depressive disorder, obesity (BMI N30), DM, opioid abuse/dependence, rheumatoid arthritis, and age at time of TKA	MUA within 90 days of TKA No definition of indication for MUA specified	Case-control	DM was not a significant risk factor for postoperative knee stiffness requiring MUA after TKA	229,420 TKAs Stiff group: Total number = 3470 Number of DM = 1050 Control group: Total number = 225,950 Number of DM = 70,280	Percentage of stiff group with DM = 30.2% Percentage of control group with DM = 31.1%
Gandhi et al. [4] 2006.	Canada	To identify the incidence of and predictive factors associated with knee flexion less than 90° at 1 year after TKA	One year postoperative flexion less than 90°	Case-control	No correlation between postoperative stiffness and specific medical comorbidities, including DM	90 TKAs Stiff group: Total number = 45 Number of DM = 8 Control group: Total number = 45 Number of DM = 3	Percentage of stiff group with DM = 17.8% Percentage of control group with DM = 6.7%
Meding et al. [12] 2003	USA	To review the results of TKR in DM patients and to test the hypothesis DM patients achieve inferior results after TKR	MUA required postoperatively. No time or indication specified	Case-control	DM not associated with an increased rate of manipulation rate after TKA. Insulin-dependent diabetics more susceptible to manipulation than noninsulin-dependent diabetic patients	5220 TKAs Stiff group: Total number = 65 Number of DM = 4 Control group: Total number = 5155 Number of DM = 325	Percentage of stiff group with DM = 6.1% Percentage of control group with DM = 6.3%

In one study [8], the CI reported did not correspond correctly to the OR given. The authors were contacted for clarification, but as this was not obtained, the reported OR and lower limit of CI along with a recalculated upper limit CI were utilized in the meta-analysis.

**Fig. 2:**
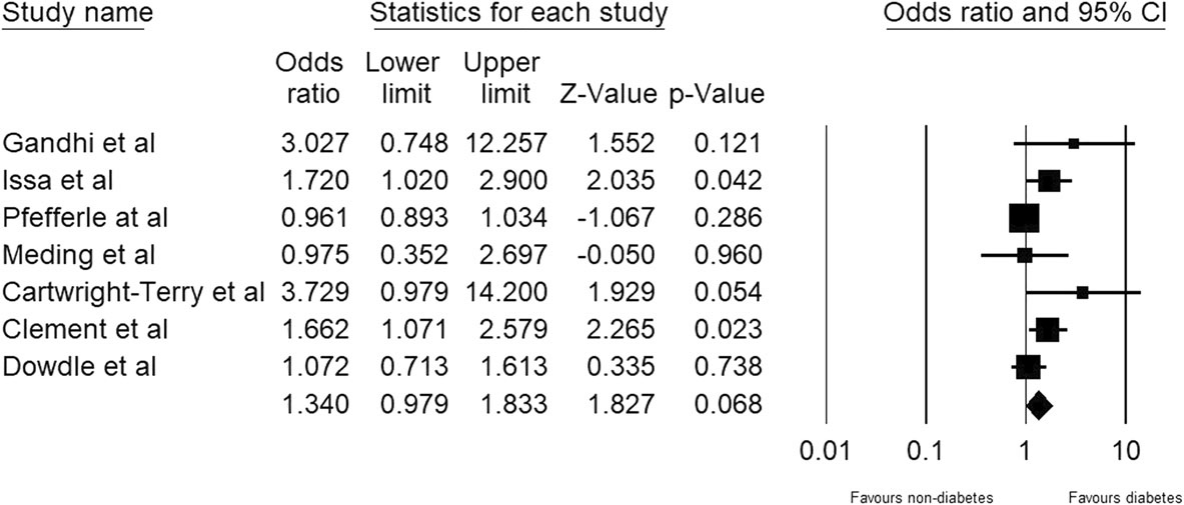
Meta-analysis of prevalence of postoperative knee stiffness after primary and revision total knee arthroplasty in populations with diabetes mellitus

**Fig. 3:**
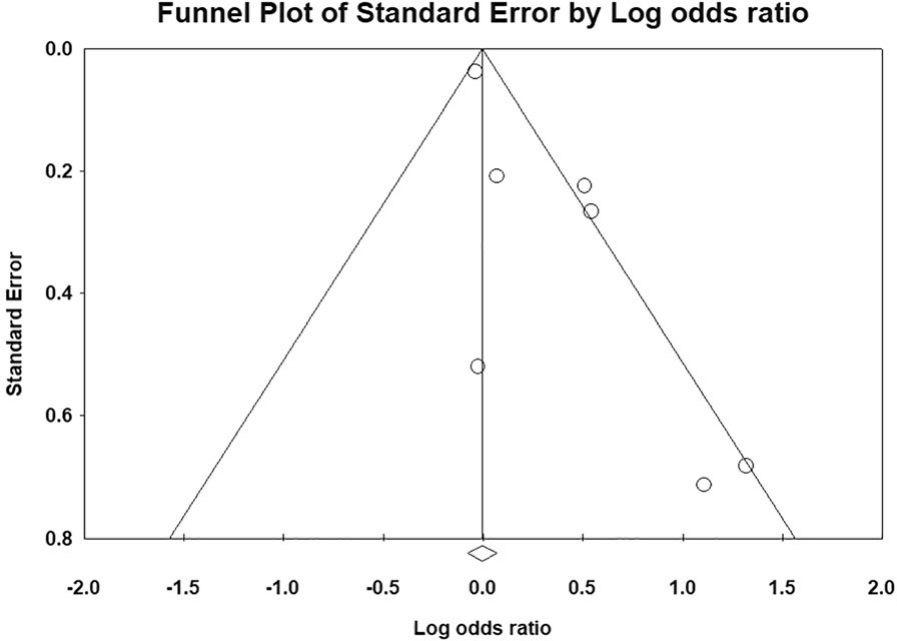
Funnel plot of prevalence of postoperative knee stiffness after primary and revision total knee arthroplasty in populations with diabetes mellitus

Meta-analysis showed that DM does not confer a higher risk of stiffness after TKA (for primary and revision TKAs combined, OR 1.340, 95% CI 0.97–1.83; for heterogeneity, tau^2^ = 0.086, I^2^ = 63.9, Q-value = 16.630, and *P* value = 0.068; Fig. 2). Funnel plot visual inspection analysis did not show an obvious small study effect (Fig. 3).

**Fig. 4:**
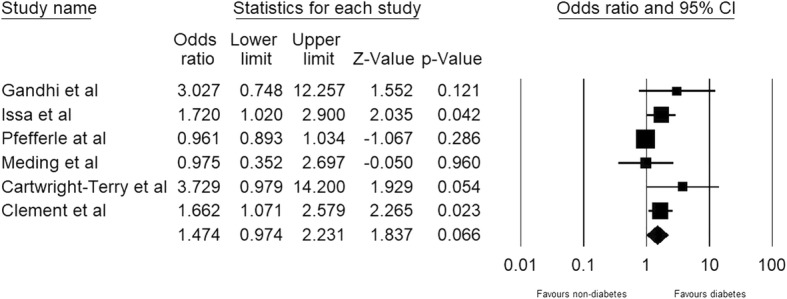
Meta-analysis of prevalence of postoperative knee stiffness after primary total knee arthroplasty in populations with diabetes mellitus

Analysis of only primary TKAs with revision TKAs excluded also demonstrates that DM does not significantly increase the risk of stiffness after primary TKA (OR 1.474, 95% CI 0.97–2.23; heterogeneity, tau^2^ = 0.143, I^2^ = 69.689, Q-value = 16.496, and *P* value = 0.066; Fig. 4).

The MINORS [15] assessment for bias showed a low score (18–22; Table 2).

**Table 2:** Quality assessment of nonrandomized studies (Methodological Index for Nonrandomized Studies)

MINORS criteria	Cartwright-Terry et al. [18] 2018	Clement et al. [17] 2018	Dowdle et al. [19] 2018	Issa et al. [8] 2015	Pfefferle et al. [16] 2014	Gandhi et al. [4] 2006	Meding et al. [12] 2003
A clearly stated aim	2	2	2	2	2	2	2
Inclusion of consecutive patients	2	2	2	2	2	2	2
Prospective collection of data	2	1	0	0	0	0	0
Endpoints appropriate to the aim of the study	2	2	2	2	2	2	2
Unbiased assessment of the study endpoint	0	0	0	0	0	0	0
Follow-up period appropriate to the aim of the study	2	2	2	2	2	2	2
Loss to follow-up less than 5%	2	0	2	1	2	2	1
Prospective calculation of the study size	1	1	2	1	2	1	2
An adequate control group	2	1	1	2	2	2	2
Contemporary groups:	2	2	2	2	2	2	2
Baseline equivalence of groups	2	2	2	1	2	2	1
Adequate statistical analyses	2	2	2	2	2	2	2
Total score	21	17	19	17	20	19	18

## Discussion

This meta-analysis shows that there is no strong evidence that diabetic patients are at an increased risk of postoperative knee stiffness compared to non-diabetic patients after TKA. Our findings suggest that fear of postoperative knee stiffness should not influence the decision to undertake TKA in diabetic patients and that diabetic patients need not be managed differently from non-diabetic patients in the postoperative period.

Diabetes is known to be associated with reduced joint mobility and adhesive capsulitis of the shoulder [13]. Studies have assessed the mechanisms underlying tissue fibrosis in DM. Increased levels of pro-inflammatory cytokines, particularly in obese patients [20], such as tumor necrosis factor alpha (TNF-α), interleukin (IL)-6, and 1B (IL-1B) [20] result in the deposition of extracellular matrix and fibrosis [21]. Recent studies also show evidence of pro-inflammatory cytokine production from Toll-like receptor 4-driven responses resulting in synovial hyperplasia, macrophage activation, cartilage catabolism, and joint destruction [22].

A range of glycated, oxidized, and nitrated proteins have been detected in the synovial fluid of patients with knee OA [23]. The formation of advanced glycation end-products (AGEs) is increased in diabetes and leads to increased crosslinking and stiffness of collagen with altered tissue function and biomechanics [24]. Increased methylglyoxal has been found specifically in the synovial fluid of diabetic compared to nondiabetic patients with OA [25]. A recent experimental study has shown that AGE increases collagen I and III gene expression only with immobilization, which is relevant to TKA [26].

Myofibroblast and growth factor numbers are known to be increased in stiff joints, including adhesive capsulitis of the shoulder and stiff elbows requiring surgical release [27]. Activation of the myofibroblast–mast cell–neuropeptide pathway in response to injury may also contribute to joint stiffness following TKA, by linking acute inflammation with subsequent contracture [28, 29]. Diabetes mellitus is known to cause crosslinking of collagen due to the formation of AGEs [24]. A recent study has shown increased expression of damage-associated molecular patterns (DAMPs), including high-mobility group box-1, the receptor for advanced glycation end products, and alarmins (S100A8 and S100A9) in the knee compared to the hip joint [30]. This is thought to facilitate increased cartilage degradation and inflammation.

From the existing literature, DM clearly has an impact on the inflammatory process, which can result in fibrosis and stiff joints, particularly in the shoulder and elbow. Results in the literature differ regarding the impact of DM on knee stiffness. This meta-analysis has shown no difference between the rate of stiffness after TKA in diabetic and nondiabetic patients. DM results in systematic inflammation and therefore it would be expected to affect all joints to a similar extent. There must be a reason as to why different joints appear to have differing susceptibilities to stiffness induced by DM. Evidence in the literature is currently inadequate to explain this variation. We postulate it may be due to different joints expressing different levels of receptors to inflammatory cells involved in the DM inflammatory cascade as described previously. The difference in structure between different joints and their frequency of use in typical daily life may also affect the risk of developing stiffness in a particular joint. Further work is needed to investigate this important difference to enhance our knowledge of the pathological process and help to provide strategies to combat joint stiffness.

A limitation of this study is that the meta-analysis included only seven studies. This highlights the limited evidence in the current literature regarding the effect of DM on postoperative knee stiffness after TKA, especially revision TKA. On the basis of the evidence currently available, our results suggest that patients with diabetes are not at an increased risk of postoperative knee stiffness compared to nondiabetic patients after TKA. The studies included in the meta-analysis reported differing results, which demonstrates the value of performing this meta-analysis in order to clarify the overall picture of results in the literature. These studies varied in their definitions of knee stiffness, and the studies included either the need for MUA or a ROM less than 90°. Most of the studies were not prospective or randomized, potentially affecting sampling bias, although they scored low on bias assessment. None of the studies stated they managed DM and non-DM patients differently, which could have affected results if, for example, more intensive physiotherapy was given to the DM patients, reducing their risk of developing stiffness. With reviews of large databases there is also the possibility of incomplete or inaccurate data. Several studies were excluded from the meta-analysis as they included both painful and stiff knees postoperatively. The majority of studies did not specify whether they included patients with type 1 diabetes, although given that the majority of patients with OA will be elderly, it is likely that they had type 2 diabetes. Furthermore, the relation between diabetes control and risk of stiffness cannot be determined.

## Conclusion

DM does not confer a higher risk of knee stiffness after TKA. We recommend that the decision to proceed with TKA, discussion as part of the consent process, and subsequent rehabilitation should not differ between patients with and without DM with regards to risk of stiffness.

## Abbreviations

ADL: Activities of daily living
AGEs: Advanced glycation end-products
BMI: Body mass index
CI: Confidence interval
CINAHL: Cumulative Index to Nursing and Allied Health Literature
DAMPs: Damage-associated molecular patterns
DM: Diabetes mellitus
EMBASE: Excerpta Medica dataBASE
IL: Interleukin 6
MINORS: Methodological Index for NonRandomized Studies
MUA: Manipulation under anaesthesia
NCBI: National Centre of Biotechnology Information
OA: Osteoarthritis
OR: Odds ratio
PRISMA: Preferred Reporting Items for Systematic Reviews and Meta-Analyses
QoL: Quality of life
ROM: Range Of movement
TKA: Total knee arthroplasty
TNF-α: Tumour necrosis factor alpha
USA: United States of America
WOMAC: Western Ontario and McMaster Universities Osteoarthritis Index
